# Efficacy of Nd:YAG Lasers for Tattoo Removal: Systematic Review of Clinical Outcomes, Clearance Rates, and Treatment Parameters

**DOI:** 10.1111/jocd.70914

**Published:** 2026-06-17

**Authors:** Jolie Jin En Wong, Sumbel Khan, Madhvi Tandel, Zaid Dajani, Ishith Seth

**Affiliations:** ^1^ Cardiff University School of Medicine Cardiff UK; ^2^ Charing Cross Hospital, Imperial College Healthcare NHS Trust London UK; ^3^ Independent Researcher London UK; ^4^ College of Medicine Imam Abdulrahman Bin Faisal University Dammam Saudi Arabia; ^5^ Department of Plastic and Reconstructive Surgery St Vincent's Hospital Melbourne Australia

**Keywords:** Fitzpatrick skin type, Nd:YAG, picosecond, pigmentary change, Q‐switched, tattoo removal

## Abstract

**Background:**

Nd:YAG lasers are widely used for tattoo removal, selectively targeting deep dermal pigment with relative melanin sparing. Both nanosecond Q‐switched and picosecond‐domain systems are employed in clinical practice.

**Objectives:**

To evaluate the efficacy and safety of Nd:YAG laser systems for tattoo removal, including clearance, treatment parameters, number of sessions, and adverse effects.

**Methods:**

A systematic review was conducted according to PRISMA 2020 guidelines and registered with PROSPERO. MEDLINE, EMBASE, Scopus, and the Cochrane Library were searched. Eligible studies were human clinical studies (≥ 10 participants) evaluating Nd:YAG laser tattoo removal, including monotherapy and accelerated or combination protocols, reporting clearance or adverse events. Risk of bias and certainty of evidence (GRADE) were assessed.

**Results:**

46 studies were included, comprising randomized controlled trials, cohort studies, and case series. Nd:YAG lasers achieved meaningful clearance of black and blue tattoo pigments in most studies, generally requiring 4–8 sessions to reach ≥ 75% clearance. Amateur tattoos responded more favorably than professional tattoos, while colored pigments demonstrated lower and more variable clearance. Picosecond Nd:YAG systems may achieve similar clearance with fewer sessions or lower fluence. However, consistent superiority over nanosecond Q‐switched Nd:YAG was not demonstrated. Adverse events were predominantly mild and transient, mainly pigmentary alteration.

**Conclusions:**

Nd:YAG lasers provide effective clearance of black and blue tattoos but require multiple sessions. Professional tattoos and colored pigments remain treatment challenges. High‐quality comparative trials with standardized outcome measures are needed to clarify the relative benefits of picosecond and nanosecond systems.

## 1 Introduction

Tattoos have become increasingly common worldwide, with prevalence estimates ranging from 10% to 30% across studies [1, 2]. As tattoos have gained cultural and aesthetic acceptance, there has also been a parallel rise in the number of people seeking removal due to professional requirements, personal dissatisfaction, or lifestyle changes [1]. This growing demand underscores the need for reliable, evidence‐based methods that can safely and effectively address unwanted pigment across a wide range of skin types.

Laser‐based treatment has become the preferred modality of tattoo removal, supplanting earlier invasive techniques such as dermabrasion and surgical excision [3]. The Q‐switched neodymium‐doped yttrium aluminium garnet laser (Nd:YAG) is widely regarded as a cornerstone technology in this context, as its 1064‐nm wavelength enables deep dermal penetration with selective melanin sparing, thereby extending its utility to both darker inks and higher Fitzpatrick phototypes [4, 5] Moreover, its frequency‐doubled 532‐nm emission extends its utility to red, orange, and yellow colored pigments [6]. More recently, picosecond Nd:YAG devices have gained popularity for their ability to deliver shorter pulse durations and greater photoacoustic effects, potentially enhancing pigment fragmentation and improving clearance rates [7, 8].

Despite widespread clinical use of Nd:YAG lasers, there remains no consensus on expected clearance, treatment burden, or the relative benefits of picosecond versus Q‐switched platforms [3, 9]. A systematic review is therefore warranted to assess the effectiveness and safety of the Nd:YAG laser for tattoo removal. This review seeks to synthesize data on clearance rates, the number of treatment sessions, laser settings, characteristics of patients and tattoos, and reported complications in data from randomized controlled trials, prospective and retrospective cohorts, and case series that have an adequate sample size [10, 11, 12].

Although multiple reviews have addressed tattoo removal using lasers, most integrate multiple laser technologies or utilize narrative summaries in the absence of a structured methodological approach [3, 9]. Growing clinical use of picosecond Nd:YAG systems, coupled with ongoing utilization of Q‐switched platforms, underscores the need for an updated, focused synthesis of available evidence [13, 14]. Clinicians lack clear guidance on whether picosecond systems offer meaningful clinical advantages, how treatment parameters influence outcomes, and how safety varies across skin phototypes [14, 15, 16].

## 2 Methods

### 2.1 Protocol and Registration

A systematic review was carried out and conducted in accordance with the Preferred Reporting Items for Systematic Reviews and Meta‐Analyses (PRISMA) 2020 guidelines and a priori registered with PROSPERO (International Prospective Register of Systematic Reviews) under registration number CRD420251241094.

### 2.2 Information Sources and Search Strategy

A comprehensive literature search was conducted in databases including MEDLINE, EMBASE, Scopus, and the Cochrane Library. Databases were searched from inception to 11 December 2025. Additional searches were performed in PubMed and ClinicalTrials.gov, the National Institute for Health and Care Excellence (NICE) evidence database, World Health Organization (WHO) reports, NHS, UK Government publications, the UK Health Security Agency, and the Nuffield Trust. Gray literature sources, including ProQuest Dissertations and Theses and MedNar, were also searched. The full search strategy for MEDLINE and EMBASE (via Ovid) is provided in Appendix S1.

“Lasers,” and “Tattoo” were used as MeSH headings to aid in the development of a search strategy. The following search strategy was used and modified as appropriate for each database: (Nd:YAG OR Q‐switched OR picosecond) AND (tattoo OR tattoo removal) AND (wavelength OR pulse duration OR fluence OR spot size) AND (clearance OR outcome OR adverse effects OR pigmentation OR scarring). The search strategy was reviewed and approved by a third author prior to execution.

### 2.3 Study Selection

Study screening was performed independently by two reviewers. Titles and abstracts were initially screened for relevance, followed by full‐text assessment of potentially eligible studies to determine inclusion. Reference lists of included studies were subsequently screened using a snowballing approach on the Rayyan platform to identify additional relevant studies not captured in the initial search. Any disagreements were resolved through discussion and consensus involving a senior reviewer.

### 2.4 Eligibility Criteria

Eligible studies were human clinical studies with a minimum sample size of 10 participants that evaluated Nd:YAG laser systems for tattoo removal, including both Q‐switched and picosecond platforms. Studies were eligible whether Nd:YAG lasers were used as monotherapy or as part of accelerated or combination protocols (e.g., multipass techniques or CO_2_‐ or Er:YAG‐assisted protocols).

Randomized controlled trials, prospective and retrospective cohort studies, and case series including at least 10 patients were eligible for inclusion. Eligible tattoo types comprised professional, amateur, cosmetic, traumatic, and medical tattoos across all Fitzpatrick skin types.

Included studies were required to report at least one clinically relevant outcome, including tattoo clearance (expressed as a percentage or using a qualitative or ordinal clearance scale), number of treatment sessions, or laser treatment parameters (e.g., fluence, spot size, or pulse duration). Studies reporting treatment‐related adverse events, side effects, or complications were also eligible for inclusion.

Studies were excluded if conducted in animals or in vitro, or if they consisted of individual case reports or case series with fewer than 10 patients. Studies evaluating laser systems other than Nd:YAG were excluded unless Nd:YAG lasers were directly assessed or compared with alternative laser platforms. Non‐clinically focused publications, including physics‐only or theoretical studies without patient outcomes, were excluded. Review articles, systematic reviews, narrative reviews, letters, opinion pieces, conference abstracts, and other non–full‐text publications were also excluded.

### 2.5 Risk of Bias in Individual Studies

The risk of bias was evaluated using validated tools appropriate to the study design. Randomized controlled trials were analyzed using the Cochrane Risk of Bias tool, observational cohort studies were examined using the Newcastle–Ottawa Scale, and case series were assessed using the Joanna Briggs Institute critical appraisal checklist. Risk‐of‐bias assessments were performed independently by two reviewers, with disagreements resolved by a third reviewer. The overall certainty of evidence was assessed using the GRADE approach. The full risk of bias assessment table is provided in Table S1.

## 3 Results

### 3.1 Study Selection

The database search identified 4224 records, and an additional 7 were found through trial registers. 3526 records were subjected to title and abstract screening after 705 duplicate records were removed. 3477 of these records were excluded for failing to meet the inclusion criteria.

A total of 47 full‐text reports were sought for retrieval and assessed for eligibility. One report was excluded at the full‐text stage as it was an individual case report and did not meet the minimum sample size requirement.

**FIGURE 1:**
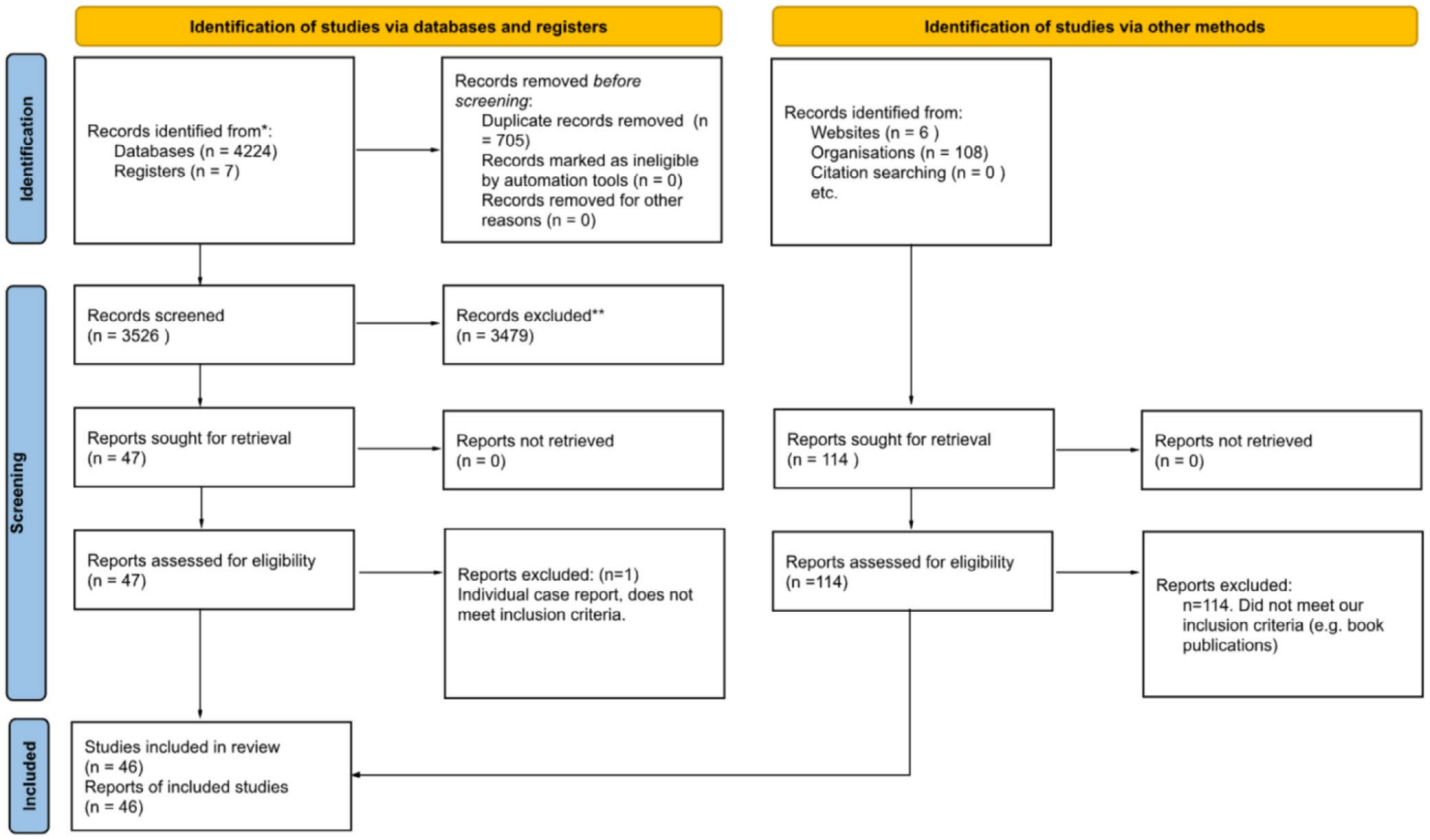
PRISMA 2020 flow diagram for new systematic reviews which included searches of databases, registers and other sources. *Consider, if feasible to do so, reporting the number of records identified from each database or register searched (rather than the total number across all databases/registers). **If automation tools were used, indicate how many records were excluded by a human and how many were excluded by automation tools. *Source:* Page MJ, et al. BMJ 2021;372:n71. DOI: 10.1136/BMJ.n71. This work is licensed under CC BY 4.0. To view a copy of this license, visit https://creativecommons.org/licenses/by/4.0/.

In addition, 114 records were identified from other sources, including websites and organizations. All were retrieved and assessed. However, none met the inclusion criteria, primarily because they were book chapters, nonclinical publications, or otherwise outside the scope of this review. As a result, 46 studies were included in the final qualitative synthesis. The study selection process is illustrated in the PRISMA 2020 flow diagram (Figure 1).

### 3.2 Study Characteristics

There are 46 studies, including randomized controlled trials, split‐tattoo comparative studies, prospective and retrospective cohort studies, and large case series. The sample sizes for the study ranged from small clinical cohorts to large retrospective cohorts or programmes involving several hundred patients.

Participants represented a broad spectrum of Fitzpatrick skin types (I–VI), with most studies including predominantly skin types III–V. Tattoo types included professional and amateur tattoos, cosmetic tattoos (e.g., eyebrow or eyeliner tattoos), traumatic tattoos, and medical tattoos. Black and blue pigments were the most frequently studied, whereas multicolored tattoos were examined in a smaller subset of studies.

Most studies evaluated Q‐switched nanosecond Nd:YAG lasers, typically at 1064 nm, with or without additional wavelengths such as 532 nm. Some recent studies have assessed picosecond‐domain Nd:YAG lasers, while several studies examined Nd:YAG lasers used in combination with accelerated treatment techniques. A summary of study characteristics is provided in Table 1.

**TABLE 1:** Characteristics of included studies.

Author	Year	Study design	Population	FST	Tattoo type	Laser type and *λ*	Comparator	NOS	Clearance outcome	Adverse events
Karsai S et al.	2008	Retro. clinical study	32P (14 M, 18 F)	NR	Prof black tattoos (resistant to prior QS Nd:YAG treatment)	1064 nm (QS Nd:YAG; MedLite C6)	NR (Before/After comparison to older C3 technology)	5	33% achieved > 75% CR	Hyper (5.6%);
Leuenberger M.L. et al.	1999	Prosp within‐tattoo comparison	42 T; P 18–75 years	NR	Blue–black tattoos (≥ 2 × 2 in.; non‐facial)	Alexandrite (755 nm), Nd:YAG (1064 nm), and Ruby (694 nm)	Within‐tattoo comparison (each tattoo divided into three sites, one for each laser)	3–6	> 95% CR (Grade 5) after 6 sessions: Ruby 38%; Alexandrite 31%; Nd:YAG 23%.	Hypo: Ruby 38%, Alexandrite 2%, Nd:YAG 0%. Hyper: Nd:YAG 7%.
Ross E. V., et al.	1998	Comparative trial (Within‐tattoo comparison)	16P (T count NR)	NR	Predominantly black tattoos; some multicolored (red, yellow, orange, blue, green)	1064 nm (QS Nd:YAG)	(35 ps) vs. (10 ns)	4	ps superior in 12/16 tattoos (75%) for black ink (*p* < 0.002).	Hypo in 1 tattoo (ps arm); no scarring except in high‐fluence ns areas
Kilmer & Anderson R.R. [17]	1993	Prosp, blinded, controlled dose–response study	25P/39 T	NR	Blue‐black and multicolored tattoos; 25 prof, 14 amat	QS Nd:YAG (1064 nm)	Dose–response (6, 8, 10, vs. 12 J/cm^2^) and prior Ruby laser resistance	4	> 75% CR in 77% of black tattoos; > 95% CR in 28% (11/39) at 10–12 J/cm^2^	Colored inks (green, yellow, red, white) were largely resistant.
Radmanesh M. & Rafiei Z. [18]	2014	Prosp split‐eyebrow comparative study	20F‐EB T	II:1, III:7, IV:12	EB cosmetic tattoos; black (7), brown (13)	Combination CO_2_ + QS Nd:YAG (1064/532 nm)	Q‐switched Nd:YAG laser alone	1	30% achieved > 75% CR with combination therapy vs. 5% with Nd:YAG alone (*p* = 0.007).	Transient erythema; post‐inflammatory hyper (2 patients)
Alster T. & Kauvar A	2015	Prosp clinical study	60P/75T	NR	Decorative tattoos; black and multicolored	ps‐domain Nd:YAG (1064 nm and 532 nm)	NR	Mean 2.9–3.8 treatments	≥ 50% CR in 89% of tattoos; ≥ 75% CR in 54% of tattoos.	Transient hyper only
Bernstein E.F. & Civiok J.M.	2013	Prosp split comparative study	12P/13T	I:1, II:6, III:4, IV:1	Decorative tattoos; 7 black‐only, 6 multicolored	QS Nd:YAG with KTP (1064 nm and 532 nm)	Largest possible spot size (MAX‐ON: 4.2–6.2 mm) vs. Standard fixed spot size (4 mm)	6	87% (MAX‐ON) vs. 86% (4‐mm beam); MAX‐ON superior through sessions 1–4 (*p* < 0.05)	NR
Narayanan S., et al.	2021	Randomized split controlled trial	35P/53T	Darker skin types (not numerically specified)	Blue/black tattoos (Thread‐like < 5 mm; Band‐like > 5 mm)	QS Nd:YAG (1064 nm; MedLite C6)	R15 method (3 passes in 1 session) vs. Conventional (1 pass per session × 3 months)	R15: 1 session; Conventional: 3 sessions	5 months (Very good/Excellent ≥ 76%): 24% (R15) vs. 27% (Conventional). Thread‐like tattoos performed better (38% R15 vs. 35% Conventional).	Blistering common in band‐like tattoos; bullae in 7 R15‐treated sides; *scarring in 3 tattoos*
Mungnirandr A., et al.	2011	Retro review study	115P (101 M, 14 F)	Darker skin types (III–IV)	Amat tattoos; black ink only; predominantly hand‐made	QS Nd:YAG (1064 nm)	Baseline (Evaluation of treatment programme)	5	76%–100% CR achieved in 75% of evaluated tattoos.	Textural changes (41%), Scarring (17%), Hyper (3%), and Hypo (3%).
Jow et al.	2010	Retro clinical study (10‐year, single center)	238P (95 M, 143 F)	(IV–VI treated with 1064 nm)	Mixed (prof, amat, cosmetic, traumatic)	QS Ruby (694 nm); QS Nd:YAG (532 and 1064 nm)	NR	Mean 3.57 sessions (range 1–17)	Total CR achieved in only 1.26% (3/238 pt).	Hypo, Hyper, pain, and bleeding (specific rates NR).
Gollamudi L.P. et al. [19]	2024	Prosp comparative study	50P	NR	Amat (68%) and prof (32%) tattoos	Comb G Ultra Pulse CO_2_+ QS Nd:YAG (1064 nm); MG: QS Nd:YAG alone	QS Nd:YAG monotherapy	Max 6 sessions (Mean for clearance: 3.58 vs. 5.86)	Combination therapy significantly faster; achieved > 95% CR in 3.58 sessions vs. 5.86 sessions for monotherapy.	Depigmentation (8.33%), Hyper (8.33%), Scarring (13.8% in CG), and Pinpoint bleeding (8.33% in MG)
Egozi et al.	2024	Retro clinical evaluation and chart review	11P	NR	Prof, amat, and traumatic tattoos (77% purely black or blue‐black)	Dual‐wavelength QS Nd:YAG (1064 nm and 532 nm)	Standard Kirby‐Desai scale predictions	3–8 treatments	High efficiency in long‐term (4–5 years) vs. short‐term (*p* < 0.001); sessions (5.09) were lower than the scale's prediction (9.9).	NR
Sravan et al.	2023	Interventional study	50P	NR	Amat (54%) and prof (46%) tattoos	QS Nd: YAG; 1064 nm (58% of pt) and 532 nm (42% of pt)	NR	7–9	More than 95% removal of the tattoo (Clear) was achieved by the end of the study.	84% had no adverse effects‐ 6% of pt. scarring was seen
Nguyen et al.	2021	Prosp clinical study	30P/50 T	III and IV	Prof 86.5% 80.8% single‐color, 19.2% multi‐colored	ps Nd:YAG (1064 and 532 nm)	Baseline	6 sessions (6–8 week intervals)	88.5% ‐“good” response (≥ 75% CR); 7.7% CR (100%).	Oedema (57.7%), burning (51.9%), bullae/blistering (25%), one case (1.9%)altered pigmentation
Kono et al.	2020	Prosp, comparative split‐lesion study	11P/37T	III to IV	Prof multi‐colored tattoos	PSL vs. NSL at 532 and 1064 nm	Split‐lesion (Internal control)	4 treatments at 4‐week intervals	PSL was more effective than NSL, for black, red, and green inks.	PIH in 21.6%–35.1% of tattoos; Paradoxical darkening in 5.4%.
Mungnirandr A. et al.	2012	Prosp comparative study (public health program)	144	III and IV	Amat tattoos (dark blue and black pigments)	QS Nd: YAG (1064 nm)	Regional comparison (Region 3 vs. Region 4)	3–5	Region 3 showed superior therapeutic outcomes compared to Region 4.	Transient hyper and pinpoint bleeding.
Pawar et al. [20]	2024	Single‐center retrospective review	22F	Type III to Type V	Cosmetic eyebrow tattoos (permanent makeup); black, brown, and reddish‐brown inks	ps Nd: YAG (1064 and 532 nm)	Baseline (Retro review)	Mean of 3.2 sessions (6–8 week intervals)	91% of pt. achieved complete or near‐complete CR (> 75% lightening).	Mild erythema, oedema, and temporary whitening of eyebrow hair.
Goyal et al.	2005	Prosp., paired comparative study (split‐tattoo)	20P(14‐prof, 6‐amat)	II–IV	12 black and 8 multicolored tattoos	QS Nd:YAG (1064 nm), QS Nd:YAG (532 nm), and QS Ruby (694 nm)	Internal paired comparison (three sections per tattoo)	1 treatment session (for comparison)	QS Ruby laser (694 nm) produced the greatest lightening in 75% of evaluations, significantly outperforming the 1064 nm Nd:YAG (23%).	*Ruby laser* caused significantly more hypo than the Nd:YAG.
Bäumler et al.	2002	Prosp., comparative split‐tattoo study	23P	NR	Black and multicolored tattoos	PSL: 532/1064 nm vs. NSL: 694 nm	Split‐tattoo (Internal control)	8 sessions at 4‐week intervals	PSL showed more effectiveness than NSL for clearance, though the difference was not statistically significant (*p* > 0.05).	PSL caused fewer and shorter‐lasting transient reactions (blistering, pruritus, burning). Hypo occurred only with NSL; no scarring was detected in either group
Bernstein et al.	2015	Prosp. clinical study	21P/31 T	I (1), II (6), III (19), and IV (1)	Black (31), green (8), red (6), blue (2), purple (2), and yellow (2)	ps Nd:YAG (1064 and 532 nm)	Baseline	10 treatments (mean not specified); interval 6–10 weeks	Average overall CR of 79% ± 0.9%. High efficacy for black (92%), yellow (85%), and red (80%).	Mild hyper (5 tattoos) and mild hypo (3 tattoos). No scarring reported.
Zhang et al.	2018	Retro. clinical evaluation	72P	III and IV	Blue‐black cosmetic eyeliner tattoos (older than 10 years)	755 nm Alexandrite ps vs. 1064 nm Nd:YAG ns	Retro comparison of two technologies	Median 1 session (Range: 1–4)	No difference in clearance between ps Alexandrite and ns Nd:YAG after a single session (*p* > 0.05).	Transient side effects only; no significant or permanent adverse reactions reported in either group.
Gold, M. [21]	2009	Prosp., randomized, split‐treatment clinical study	14P (8 M, 6 F)	NR	black and dark blue ink (> 50%); 7 prof, 1 amat, 3 unclassified	EO QS Nd:YAG (1064 nm primary; 532, 650, 585 nm)	SP vs. Pulse Dispersion in PTP modes	4 monthly treatments	SP‐treated achieved better results (36.4% “excellent” clearance > 76%) compared to the PTP‐treated side (primarily “fair” to “good” improvement).	Erythema (44.8%) and oedema (41.4%).
Kato et al.	2020	Single‐center retro study	42P	III, IV	Black ink tattoos	1064 nm and 532 nm Nd:YAG (PSL &NSL)	comparison (to traditional QS Nd: YAG monotherapy)	Mean 3.8 sessions for 2 ns; Mean 3.1 sessions for 750 ps	combination of 2 ns and 750 ps pulse widths removes tattoos in significantly fewer sessions than traditional QS laser treatment.	NR
Nibras A. A. Hindy [22]	2020	Case Series	176P/293T (136‐amat, 40‐prof)	III, IV	Amat and Prof; Black, blue, and red inks	QS Nd: YAG (1064 nm and 532 nm)	Amat vs. Prof CR	1–5 sessions (Prof required significantly more)	Amat tattoos achieved 95% CR in 70% of cases; Prof tattoos achieved 95% clearance in 50% of cases.	Hypo reported.
Kaminer et al.	2020	Single‐center prosp. trial (Split‐tattoo design)	21P	I–III	Prof	1064 nm QS Nd:YAG	Single‐pass Laser (Conventional) vs. Multi‐pass Laser + Rapid Acoustic Pulse (RAP) device	1 session (Evaluation of immediate fading)	RAP‐assisted side allowed for an average of 4.15 passes in a single session, resulting in significantly greater fading compared to the single‐pass conventional side.	Erythema, oedema, and crusting.
El‐Domyati et al.	2019	Clinical trial with histometric analysis	12P	III–V	Blue tattoos	1064 nm QS Nd:YAG	Baseline clinical and histological status	Mean 7.5 sessions (Range: 6–10)	66.7% pt. show 75%–100% CR, mean improvement of 77.9%.	Mild erythema was the only reported complication
Aurangabadkar et al.	2019	Open‐label prosp clinical study	22‐Amat. T	IV and V	Black amat tattoos	QS Nd:YAG (1046 nm)	Historical/Standard single‐pass (Correlation with Kirby‐Desai scale)	1–4 sessions	81.8% achieved ≥ 90% CR; 95.4% achieved ≥ 75% CR.	Depigmentation, mild scarring, and ghosting
Padhiar et al.	2019	Randomized, prosp interventional study	89P; Mean age 21.9 years	III–V	Amat (70.8%) and Prof (29.2%); Blue‐black (92.1%), Red, and Green (7.9%)	QS Nd:YAG (1064 nm and 532 nm)	Fluence comparison (7 J/cm^2^ vs. 9 J/cm^2^)	Range of 4–9 sessions (13.5% of patients required ≥ 10 sessions)	Amateur blue‐black tattoos showed significantly better CR at higher fluence (91.0% vs. 54.3% achieving ≥ 76% clearance; ≤ 0.02). Prof tattoos responded poorly, with 0% achieving ≥ 76% clearance.	*Immediate:* Pain (74.2%), Erythema/Ooedema (68.5%), Pinpoint bleeding (11.2%). *Early:* Burns/Blisters (2.2%). *Delayed:* Hypo(12.4%), Hyper (6.7%).
Vangipuram et al.	2018	Retro, single‐institution chart review	14P (Ages 23–44)	IV–VI	Black, blue, red, green, purple, and pink tattoos	QS Nd: YAG (1064 nm) and ps Nd:YAG (532, 785, 1064 nm)	N/A (Safety evaluation of PFD patch use across different laser types)	1 to ≥ 2 sessions (71% underwent ≥ 2)	NR	NR
Lorgeou et al.	2018	Prosp randomized study	49P (71.4% NSL treatment)	III–IV	Prof tattoos (85.7%); Black/blue (89.8%) and Polychromatic (10.2%)	1064 nm and 532 nm (comparing 2 PSL vs. 1 NSL)	Nanosecond Nd:YAG (5 ns)	1–4 sessions	PSL showed > 75% CR in 33% of tattoos, compared to 14% for the NSL.	Pain reported during treatment.
Biesman and Costner	2017	Prosp randomized single‐site split‐tattoo study	30P/30 T	I–IV	Prof black/blue tattoos	755 nm (QS Alexandrite)	Split‐tattoo: Laser with PFD patch vs. Laser without patch	1session (Pivotal trial for device efficiency)	PFD patch allowed more laser passes (Mean 3.7) vs. control (Mean 1.4) within a 5‐min window.	Transient erythema and oedema, PFD patch side showed fewer adverse events than the control side.
Pinto et al.	2016	Randomized controlled, single‐blind clinical trial	21P/30T	NR	Prof applied black tattoos only	1064 nm Nd:YAG ps vs. ns	Nanosecond Nd:YAG (5 ns)	2 sessions (6‐week interval)	Median CR 25%–36%; no difference between ps and ns after 2 sessions	Transient blistering, crusting, and swelling, Hypo and hyper reported
Sardana et al.	2015	Prosp split‐lesion clinical study	10P	NR	5 amat and 5 prof tattoos; predominantly blue–black ink	1064 nm (QS Nd: YAG) and 2940 nm (Er:YAG)	Split‐lesion: QS Nd:YAG alone vs. Er:YAG + QS Nd:YAG combination	*Combination*: Mean 1.6 (amat) to 4 (prof) *Nd:YAG alone*: 3–5 (amat) to 10 (prof)	The combination side achieved ≥ 76% improvement significantly more rapidly than the Nd:YAG monotherapy side.	Transient erythema and crusting. Mild hypo (resolved within 4 weeks)
Ahčan et al.	2013	Retro case series	11P/13T	NR	Mixed amat and prof tattoos (black, blue, green, red, cosmetic, and multicolored)	QS Nd:YAG (1064 nm) with KTP (532 nm) and dye handpieces (650 nm, 585 nm)	Baseline	3–21 sessions (Mean < 7)	Satisfactory removal was achieved in all 11 pt.	Mild erythema, oedema, and occasional lymphadenopathy.1 P paradoxical tattoo darkening
Boehncke et al.	1994	Prosp within‐individual comparative clinical study	31P (25M, 6F)	NR	Monochromatic black, amateur (non‐prof) tattoos; located on arms	QS Nd:YAG (1064 nm) vs. QS Alexandrite (755 nm)	Alexandrite 755 nm	5 sessions; 14‐day intervals	Nd:YAG laser was superior in all comparisons. In the Prism group, Nd:YAG achieved 4 complete and 8 major removals, while Alexandrite achieved 0 complete and only 4 major removals.	Transient erythema, ooedema, and occasional transient textural changes.
Reilly et al.	2023	Single‐center retro chart review	502P/2118T (56% M, 44% F);	Majority III–IV (84% of reported cases)	Monochromatic (black/blue) and colored (red, green, yellow); Prof and amat	Pulse dye laser machines (Varied based on ink color; specific wavelengths NR)	Internal comparison of complication rates vs. fluence and tattoo type	Tattoos treated 3–65 times (most patients 3–10 times)	163 of 2118 tattoos completed. Completion rate was higher in patients without complications (14.4%) vs. those with complications (7.3%).	118/2118 tattoos (5.57%) had ≥ 1 complication including hypo, hyper, keloids, and scarring.
Ross et al.	2016	RCT	10P/20 T	I–VI	Black/Dark Blue ink; ≥ 1 year old (Excluded: tribal, scarred, high‐ink density/highly colorful tattoos)	Nd:YAG (1064 and 532 nm)	Picosecond vs. Nanosecond pulse durations	NR (Outcomes tracked at 12 weeks post‐treatment)	Mean PGA at 12 weeks: ps (3.0) vs. ns (3.0) on a 4‐point scale.	Erythema (100%), oedema (100%), and Purpura (50%) in both treatment arms.
Sirithanabadeekul P., et al.	2022	Split‐Tattoo RCT	11P/19T (13‐T completed)	III (42.10%), IV (42.10%), and V (15.79%)	black tattoos (18/19); 57.89% Prof; located on trunk or extremities	1064 nm ps Nd:YAG laser	Unfractionated Picosecond laser alone vs. Combined (Unfractionated + Fractionated Picosecond laser)	3 sessions (4‐week intervals); Follow‐up at week 12	The combined group showed significantly better results. At week 12, ≥ 50% CR was achieved in 84.6% of the combined group vs. 69.2% in the ps‐only group (*p* < 0.05).	Blistering in the combined group (21.27% of sessions) vs. ps‐only group (31.91%). Textural changes in 3 P; mild hypo 1 P;
Abbas et al.	2021	Prosp Comparative Trial	40P/40T	II, III, and IV (Majority)	20 Prof, 20 Amat; Black, blue, green, and red ink	1064 nm QS Nd:YAG laser	Prof vs. Amat tattoo response to multi‐pass technique	3 sessions (3‐week intervals)	Amat tattoos showed higher CR (Mean 35%) compared to Prof tattoos (Mean 15%) after 3 m	Small blisters, purpuric rash, and transient inflammation.
Bennardo et al.	2021	Single‐center retro study	34P (17 F, 17 M)	II (29.4%), III (38.2%), and IV (32.4%)	Prof (44.1%), Amat (26.5%), Cosmetic (11.8%), Traumatic (11.8%), Medical (5.9%); Dark, red, or mixed tonalities.	1064 nm and 532 nm (QS ps)	NR	Mean 3.3 ± 2.0 sessions (Maximum 7 sessions)	41.2% P achieved complete CR (defined as 80–100% removal). Notably, all patients achieved at least a 60% removal rate.	transient petechiae‐ 6P, final “ghost effect” (hypo silhouette)‐ 3P
Cannarozzo et al.	2021	Double‐center retro study	52P (30‐F, 22‐M)	II (30.8%), III (28.8%), and IV (40.4%)	Prof (48.1%), Amat (17.3%), Medical (13.5%), Traumatic (13.5%), and Cosmetic (7.7%); predominantly dark, red, or mixed colors	QS Nd:YAG (1064 nm and 532 nm)	NR	Mean 4.6 ± 2.5 sessions (Maximum 9 sessions)	51.9% P achieved complete CR (80%–100% removal). Notably, 100% of the cohort achieved at least a 60% removal rate.	Transient petechiae‐ 7P, final “ghost effect” (hypo silhouette)‐2 P;
Priya et al.	2018	Prosp Interventional Single Centre Study	40P (25 M, 15 F)	V (70%); IV (25%); III (5%)	Amat 92.5%& Prof 7.5%; Colors: Green 57.5% & Black 40%	1064 nm (QS Nd:YAG)	Baseline/Amat vs. Prof response	Mean 8.22 ± 2.04 sessions (Max 15 sessions)	100% of all tattoos achieved 85–100% CR by the end of the study.	Frosting 100%, Ooedema 100%, Erythema 30%, Hypo 22%, Textural changes 26%, & Scarring 13%.
Mayada A. et al.	2024	Prosp simple randomized study (Single Center)	20P	IV	Prof black tattoos	1064 nm ps Nd: YAG (PicoWay)	NR	2 sessions (8 weeks apart)	Mean improvement was 61% ± 24.6%. 40% of the cohort show “Excellent” improvement (> 75% CR).	Erythema (100%) and Petechiae (100%).
Wai S.H., et al.	2006	Prosp RCT	55P (31‐M, 24‐F)	III–V	Prof blue ‐black tattoos	1064 nm QS Nd:YAG	Use of onion extract, heparin, allantoin gel vs. control for scar prevention	Mean 5.4 ± 1.3 sessions (range 4–8)	Mean 80.4 ± 11.3% CR (60% –100%)	Scarring‐23.5% (16/68 tattoos); hypo‐7.2% (4 pt), Hyper‐9% (5P).
Kauvar et al.	2017	Prosp Self‐controlled clinical study	34P/39T	NR	95% Prof; 56% single‐colored, 44% multi‐colored	1064 nm and 532 nm ps Nd:YAG (PicoWay)	Baseline/Self‐controlled	Mean 7.5 sessions (Maximum 10); Interval: 4.8 ± 1.6 weeks	After 3 treatments, 86% achieved ≥ 50% CR. After up to 10 treatments, 92% achieved ≥ 75% CR	Pruritus (8P), Mild textural change (4 P), mild hyper (1 P)
Lakshmi C. et al.	2015	Prosp Interventional Study	12P/12 T	III–VI	10‐Amat, 2‐Prof; All blue‐black ink	1064 nm QS Nd:YAG (MedLite C6)	NR	3 sessions for initial (4–6 week intervals). Full CR was observed to require 6 sessions for amat and 8–10 sessions for prof tattoos.	After 3 sessions, the average improvement was 64.1% (GAS). No patients achieved complete CR within the first 3 sessions.	Minimal erythema in all patients; 1 blister over a “double tattooed” area.

Abbreviations: Comb G, combination group; CombG, combination group; CR, clearance rate; EB, eyebrow; EO, electro‐optic; Er, erbium; F, female; FST, Fitzpatrick skin type; Hyper, hyperpigmentation; Hypo, hypopigmentation; I–IV, Fitzpatrick types I–IV; M, male; MG, monotherapy group; NOS, number of sessions; NR, not reported; ns, nanosecond; NSL, nanosecond laser; P, patients; PFD, perfluorodecalin; PIH, post‐inflammatory hyperpigmentation; Prof./Amat., professional/amateur; Prosp, prospective; ps, picosecond; PSL, picosecond laser; PTP, photoacoustic therapy pulse; QS, Q‐switched; RCT, randomized clinical trial; Retro, retrospective; SP, standard pulse; T, tattoos; *λ*, wavelength.

### 3.3 Tattoo Clearance Outcomes

Across included studies, Nd:YAG laser treatment was consistently associated with substantial tattoo lightening, particularly for black and blue pigments. Using a ≥ 75% clearance threshold, most studies reported substantial clearance in a majority of treated tattoos following multiple treatment sessions.

The number of treatment sessions required to achieve higher levels of clearance varied across studies, ranging from single‐session protocols in some comparative designs to extended treatment courses of more than 10 sessions in several cohorts. Amateur tattoos tended to clear more rapidly than professional tattoos, whereas colored pigments showed lower and more variable clearance rates. Near‐complete clearance (≥ 90%–95%) was reported in a subset of studies, typically after more treatment sessions.

### 3.4 Subgroup Analyses by Comparator

#### 3.4.1 Picosecond Versus Nanosecond Nd:YAG Lasers

Comparative studies evaluating picosecond‐domain Nd:YAG lasers versus conventional nanosecond Q‐switched Nd:YAG systems demonstrated comparable, and in some studies improved, clearance outcomes with picosecond lasers, particularly for black and dark blue tattoos. Several studies reported similar clearance with fewer treatment sessions or lower fluence settings when using picosecond lasers. However, not all studies demonstrated statistically significant differences in clearance, particularly in studies with limited treatment sessions or short follow‐up periods.

Across these studies, picosecond Nd:YAG lasers were typically associated with reduced treatment‐related pain and improved tolerability compared with nanosecond systems, as evaluated by patient‐reported pain and tolerability outcomes.

#### 3.4.2 Nd:YAG Versus Alternative Laser Systems

Within‐tattoo and split‐lesion comparative studies assessed Nd:YAG lasers against alternative laser systems, most commonly ruby and alexandrite lasers. Nd:YAG lasers demonstrated comparable or higher clearance for black and blue tattoos, with lower rates of pigmentary side effects. While ruby lasers achieved high clearance in some studies, they were associated with higher rates of hypopigmentation. Alexandrite lasers showed variable efficacy and were generally less effective than Nd:YAG lasers for monochromatic black tattoos.

#### 3.4.3 Nd:YAG Monotherapy Versus Accelerated or Combination Approaches

Several studies compared Nd:YAG laser monotherapy with accelerated or combination approaches, including CO_2_‐assisted or Er:YAG‐assisted Nd:YAG treatment, fractional picosecond delivery, and multipass protocols. These approaches were primarily designed to reduce the number of treatment sessions required to achieve comparable clearance.

Across these studies, accelerated or combination techniques were associated with faster tattoo fading and fewer clinic visits. However, increased rates of transient adverse effects, such as blistering, crusting, or short‐term pigmentary change, were reported in some studies, particularly with more aggressive or multipass protocols.

### 3.5 Laser Treatment Parameters

Laser treatment parameters varied widely across studies. For 1064‐nm Nd:YAG lasers, reported fluence values ranged from low to moderate, with spot sizes approximately 0.6–10 mm (most commonly 2–6 mm). Pulse durations reflected the laser platform used, ranging from nanoseconds for Q‐switched systems to picoseconds for newer picosecond‐domain devices. Treatment intervals were most often set at 4–8 weeks, with some studies adopting longer intervals during later stages of treatment.

Owing to heterogeneity in reporting and study design, direct comparison of optimal laser parameters across studies was not feasible. Reported laser treatment parameters are summarized in Table 2.

**TABLE 2:** Laser treatment parameters reported in included studies.

Author	Year	Fluence (J/cm^2^)	Spot size	Pulse duration	Treatment interval	Passes
Karsai S. et al.	2008	Mean Emax 6.4 ± 1.6 J/cm^2^	Mean 5.0 ± 0.9 mm (range 3.6–7.6 mm)	8–10 ns	4‐week intervals	NR
Leuenberger M.L. et al.	1999	Alexandrite: 6–8 J/cm^2^; Nd:YAG: 5–10 J/cm^2^; Ruby: 4–10 J/cm^2^	Alexandrite: 3.0 mm; Nd:YAG: 3.0 mm; Ruby: 5.0 mm	Alexandrite: 50–100 ns; Nd:YAG: 10–20 ns; Ruby: 25–40 ns	6–7 week intervals	NR
Ross E. V., et al.	1998	0.65 J/cm^2^ (EG); 8.0 J/cm^2^ (CG)	1.4 mm (ps); NR ns arm	35 (ps) vs. 10 (ns)	4‐week intervals	NR
Kilmer S.L., et al.	1993	6.0, 8.0, 10.0, and 12.0 (EG)	2.5 mm	10 ns	3–4 week intervals	NR
Radmanesh M. & Rafiei Z.	2014	CO_2_: 4 (1st pass), 3.2 (2nd pass); Nd:YAG: 7 (1064 nm), 3 (532 nm)	3 mm (Nd:YAG); CO_2_ spot size NR	Nd:YAG: 5 ns; CO_2_: NR	Single session study	CO_2_: 2 passes; Nd:YAG: 1 pass
Alster T. & Kauvar A	2015	NR	NR	Picosecond‐domain (Exact duration NR)	NR	NR
Bernstein E.F. & Civiok J.M.	2013	1064 nm: 4.2–9.2 J/cm^2^; 532 nm: 1.8–2.6 J/cm^2^	MAX‐ON: 4.2–6.2 mm; MAX‐OFF: 4.0 mm	NR	2‐month (8‐week) intervals	Single pass per session
Narayanan S., et al.	2021	5 J/cm^2^	4 mm	NR (MedLite C6 standard is 5–20 ns)	R15: 15 min between passes; Conventional: Monthly	R15: 3 passes; Conventional: 1 pass
Mungnirandr A., et al.	2011	3.5–7.5 J/cm^2^ (Mean≈5.4–5.6 J/cm^2^)	3 mm	6 ns	2‐month (8‐week) intervals	NR
Jow et al.	2010	Mean: 1064 nm = 4.11; 532 nm = 2.8; Ruby = 4.04	4‐5 mm	NR	NR	NR
Gollamudi L.P., et al.	2024	Comb G‐2.92 ± 0.77 (YAG arm); MG 6.20 ± 1.19	NR	NR	6 week intervals	Combination: 1 pass CO2 followed by 1 pass QS Nd:YAG.
Egozi et al.	2024	NR	NR	Short‐pulsed QS modality	2–3 months	Single pass
Sravan et al.	2023	NR	NR	NR	4 weeks	NR
Nguyen et al.	2021	1064 nm: 2.70–2.99 J/cm^2^; 532 nm: 1.05–1.12 J/cm^2^	3 mm to 6 mm	450 ps (for 1064 nm) and 375 ps (for 532 nm)	6–8 weeks	Single pass
Kono et al.	2020	PSL: ~2.3–3.4 J/cm^2^; NSL: ~5.5–7.0 J/cm^2^	3 mm	450–750 ps vs. ns	4 weeks	Single pass (implied)
Mungnirandr A. et al.	2012	2.5 and 5.0 J/cm^2^	2–3 mm	ns range (QS)	NR	NR
Pawar et al.	2024	1064 nm: 0.8–2.4 J/cm^2^; 532 nm: 0.3–0.6 J/cm^2^	2 mm and 4 mm	450–600 ps	6–8 weeks	Single pass (implied)
Goyal et al.	1997	1064 nm: 5.0; 532 nm: 2.0; Ruby (694 nm): 7.0–8.0	Nd:YAG: 3.0 mm; Ruby: 5.0 mm	Nd:YAG: 10–20 ns; Ruby: 25 ns	N/A (Single session comparison)	Single pass
Bäumler et al.	2002	NR	NR	PSL vs. NSL	4 weeks	NR
Bernstein et al.	2015	1064 nm: 1.4–5.3 J/cm^2^; 532 nm: 0.4–2.1 J/cm^2^	3 mm to 5 mm	450 ps (1064 nm) and 350 ps (532 nm)	6–10 weeks	Single pass
Zhang et al.	2018	PSL: 1.96–6.37 J/cm^2^; NSL: 2.80–7.00 J/cm^2	PSL: 2.0–3.6 mm; NSL: 3 mm	PSL: 750 ps; NSL: 5–20 ns	NR	NR
Gold, M.	2009	3.2–3.6 J/cm^2^	6‐mm (SP mode) and 8‐mm (PTP mode)	QS ns with unique pulse dispersion (PTP)	4 weeks (monthly)	2–3 passes per session for dark ink
Kato et al.	2020	1064 nm: 1.2–4.3 J/cm^2^ (ns); 0.5–7.0 J/cm^2^ (ps) 532 nm: 0.3–1.2 J/cm^2^	2–8 mm	2 ns and 750 ps	NR	NR
Nibras A. A. Hindy	2020	NR	3 mm	< 15 ns	NR	NR
Kaminer et al.	2020	Laser + RAP: 5.22 J/cm2; Laser only: 3.9 J/cm2	4 mm	NR	N/A (Single session trial)	Laser + RAP: Mean 4.15 passes; Laser only: 1 pass
El‐Domyati et al.	2019	5–10 J/cm^2^	3–5 mm	NR	NR	NR
Aurangabadkar et al.	2019	5–6 J/cm^2^	5 mm	2–5 ns	NR	Accelerated multipass (R0 technique utilizing PFD)
Padhiar et al.	2019	7 and 9 J/cm^2^	3 mm (1064 nm) and 2 mm (532 nm)	NR	4 weeks	NR
Vangipuram et al.	2018	0.25–12.0 J/cm^2^ (Maximum tolerated fluence)	3–10 mm	NR	NR	Up to 3 passes per session (Enabled by PFD patch)
Lorgeou et al.	2018	2–8.4 J/cm^2^ (device dependent)	NR	450–750 ps vs. 5 ns	NR	NR
Biesman and Costner	2017	4.0–5.0 J/cm^2^	4 mm	NR	N/A (Single session trial)	Mean 3.7 passes (with PFD patch) vs. 1.4 passes (without patch)
Pinto et al.	2016	ns: 3.8–11.0 J/cm^2^; ps: 1.1–4.6 J/cm^2^	ns: 3–8 mm; ps: 2–10 mm	Nanosecond: 5 ns; Picosecond: 450 ps	6 weeks	Single‐pass per session
Sardana et al.	2015	Nd:YAG: ~7–12 J/cm^2^ (adjusted to produce whitening) Er:YAG: 7–12 J/cm2	Nd:YAG: 1–4 mm; Er:YAG: 1–6 mm	Nd:YAG: 6 ns; Er:YAG: Short‐pulsed (exact duration NR)	NR	2–4 Er:YAG passes (epidermal ablation) followed by a single Nd:YAG pass.
Ahčan et al.	2013	2–9 J/cm^2^ (Mean~6 J/cm^2^; progressively increased)	2–6 mm (Most commonly 5 m)	ns(QS)	NR	Single pass per session (until whitening endpoint)
Boehncke et al.	1994	Nd:YAG: 9 J/cm^2^ (fiber), 4 J/cm^2^ (prism) Alexandrite: 18 J/cm2 (fiber), 4 J/cm2 (prism)	0.6 mm (fiber); 1.8 mm (prism)	Nd:YAG: 10 ns; Alexandrite: 100 ns	14 days	NR
Reilly et al.	2023	Mean values not specified; complication threshold reported by quarters (Q1: up to 1.44 J/cm^2^; Q4: up to 4.58 J/cm^2^)	NR	NR	NR	NR
Ross et al.	2016	NR	NR	ps vs. ns	NR	NR
Sirithanabadeekul P., et al.	2022	Unfractionated‐pico: 1.5–7.24 J/cm^2^; Fractionated‐pico: 0.8 J/cm^2^ (fixed)	3.0–4.5 mm (for unfractionated treatment)	ps	4 weeks	Combined group received both fractionated and unfractionated passes
Abbas et al.	2021	10 J/cm^2^ (Fixed)	3–5 mm (Majority 3 mm)	8–100 ns	3 weeks	Multi‐pass (specific number per session not detailed, but utilized a multi‐pass protocol)
Bennardo et al.	2021	1064 nm: Up to 5 J/cm^2^; 532 nm: Up to 3 J/cm^2^	NR	1064 nm: 450 ps; 532 nm: 370 ps	8‐week interval for the first 4 sessions, then increased to 12 weeks.	NR
Cannarozzo et al.	2021	1064 nm: Up to 10 J/cm^2^; 532 nm: Up to 5 J/cm^2^	NR	6 ns (for both 1064 nm and 532 nm)	Minimum 8‐week interval; increased to 12 weeks after the 4th session	NR
Priya et al.	2018	8–10 J/cm^2^ (Started at 8, increased by 0.2 J/cm^2^ per session)	4 mm	ns	Mean 8.22 ± 2.04 sessions	NR
Mayada A. et al.	2024	4–5 J/cm^2^	2/3 mm	450 ps	8 weeks	NR
Wai S.H., et al.	2006	3.6–4.8 J/cm^2^ (Mean 4.1 ± 0.7)	3 mm	6 ns	NR	NR
Kauvar et al.	2017	1064 nm: 0.7–8.5 J/cm^2^; 532 nm: 0.3–1.9 J/cm^2^	2 mm to 6 mm	1064 nm: 450 ps; 532 nm: 375 ps	~5 weeks (4.8 ± 1.6 weeks)	NR
Lakshmi C., et al.	2015	1.8–9.0 J/cm^2^ (Fluence adjusted based on skin type; Types V–VI were started at a lower threshold of 3 J/cm^2^).	3–6 mm (4 mm most common).	ns	4–6 weeks.	NR

Abbreviations: CG, control group; CombG, combination group; EG, experimental group; Er, erbium; KTP, potassium titanyl phosphate (KTiOPO_4_); NR, not reported; PFD, perfluorodecalin.

### 3.6 Number of Passes

In many conventional Nd:YAG laser protocols, treatment was delivered in a single pass per session. In contrast, accelerated or multipass techniques involve multiple passes within the same session or repeated passes over short intervals. These approaches were intended to reduce the total number of clinic visits, rather than to increase the clearance achieved in a single session. The number of passes was summarized descriptively where reported and was not analyzed as a primary outcome.

### 3.7 Adverse Events and Safety Outcome

Adverse events were reported in the majority of studies and were predominantly mild and transient. Common immediate effects included erythema, edema, frosting, blistering, and crusting. Pigmentary changes, including hypopigmentation and hyperpigmentation, were among the most frequently reported complications and appeared to vary according to skin type, fluence, and treatment technique.

Persistent scarring and long‐term textural changes were uncommon and were reported in only a minority of studies, particularly those using higher fluence settings, combination therapies, or aggressive multipass approaches. Overall, Nd:YAG lasers demonstrated a favorable safety profile, particularly in individuals with darker skin types, when compared with some alternative laser systems.

### 3.8 Patient‐Reported Outcomes

Patient‐reported outcomes were inconsistently reported across studies and were assessed using non‐standardized self‐reported measures, most commonly visual analogue scales or satisfaction questionnaires. Where assessed, overall patient satisfaction was generally high, particularly in studies achieving substantial clearance with fewer treatment sessions. Pain scores were often lower in picosecond Nd:YAG treatment arms compared with nanosecond systems.

### 3.9 Synthesis Approach

Given the substantial clinical and methodological heterogeneity across studies, including differences in study design, clearance assessment methods, laser parameters, and follow‐up duration, quantitative meta‐analysis was not feasible. Findings were therefore synthesized narratively.

### 3.10 Quality Assessment

Using the GRADE framework, the overall certainty of evidence for Nd:YAG laser tattoo removal across the 46 included studies was rated as low to very low. The evidence was downgraded for risk of bias, as most studies were non‐randomized observational cohorts or case series, and several randomized or split‐tattoo comparative trials had methodological limitations including unclear randomization procedures, lack of allocation concealment, incomplete blinding of outcome assessors, and attrition during follow‐up. Inconsistency was present due to substantial heterogeneity in study design, tattoo characteristics (professional vs. amateur, pigment composition, anatomical site), laser platforms (nanosecond vs. picosecond), treatment regimens (including multipass/accelerated approaches), and clearance assessment methods, with outcomes reported using nonuniform clearance scales and clearance thresholds (e.g., ≥ 75% vs. ≥ 90%–95%). The certainty of evidence was further downgraded for imprecision, as many studies had small sample sizes and reported wide variability in clearance rates and adverse event frequencies. Indirectness was identified in subsets of studies focusing on selected populations (e.g., cosmetic tattoos only, previously treatment‐resistant tattoos) or adjunctive/accelerated protocols that may not reflect routine clinical practice. Publication bias could not be excluded given the predominance of small single‐center studies and the inclusion of limited‐report evidence (e.g., registry/abstract‐style reporting). Despite these limitations, the direction of effect across studies consistently supported meaningful clearance of black and blue‐black tattoos with Nd:YAG lasers and a generally favorable safety profile with mostly transient adverse events; however, confidence in precise effect estimates, particularly for colored pigments, darker skin phototypes, and accelerated protocols, remains limited.

## 4 Discussion

This systematic review synthesizes the available clinical evidence evaluating the efficacy and safety of Nd:YAG laser systems for tattoo removal across a broad range of patient populations, tattoo characteristics, and laser platforms. Overall, Nd:YAG lasers remain the first‐line modality for the removal of black and blue tattoo pigments across Fitzpatrick skin types, with a generally favorable safety profile, including in cohorts with darker phototypes [4, 10, 23]. However, significant variability across study designs, outcome measures, and treatment protocols limits confidence in precise effect estimates and precludes firm conclusions regarding optimal treatment parameters [3, 14].

Comparative studies evaluating picosecond‐domain versus nanosecond Q‐switched Nd:YAG lasers suggest that picosecond systems may achieve comparable clearance with fewer treatment sessions or lower fluence settings, particularly for dark pigments [7, 13, 24]. These findings were not uniform across studies, and differences were less apparent in trials with limited treatment sessions or short follow‐up durations [7, 25, 26]. While picosecond technology may reduce treatment burden, its clinical superiority over conventional Q‐switched systems remains unproven and cannot be established with certainty given the current paucity of evidence [9, 12, 27].

The potential advantage of picosecond‐domain lasers lies in their shorter pulse durations, which may better match pigment relaxation times and enhance photoacoustic fragmentation, thereby delivering energy more efficiently into pigment fragmentation via a predominantly photoacoustic rather than photothermal mechanism [28, 29]. Finer pigment fragmentation may facilitate faster clearance via immune‐mediated pathways, including macrophage uptake and downstream lymphatic transport [30, 31], which may explain why some studies reported comparable clearance with fewer treatment sessions rather than higher clearance rates. In addition, reduced thermal diffusion to surrounding tissue may account for the lower pain scores and favorable tolerability reported in some comparative studies [13, 32, 33]. However, the clinical significance of these theoretical advantages remains unclear, as not all studies have shown a significant reduction in treatment sessions or enhanced clearance rate [7, 9, 25].

Laser treatment parameters varied widely across studies, reflecting differences in laser platforms, operator preference, and patient or tattoo characteristics [34, 35, 36]. This heterogeneity in parameter reporting and study design precluded identification of optimal treatment settings and highlights the absence of standardized protocols in clinical practice [16, 36]. Accelerated or multipass techniques may offer greater treatment efficiency, with more rapid tattoo fading and fewer treatment sessions in some studies [37, 38, 39]. However, certain accelerated or multipass protocols have been reported to have higher frequencies of transient local adverse effects [38, 40, 41]. The addition of adjuvants, such as perfluorodecalin‐infused patches, was investigated to improve safety and expedite these intensive protocols [42, 43].

Although Nd:YAG laser treatment demonstrates general effectiveness for dark pigments, the presence of colored inks or professional tattoos has consistently been identified as a significant limitation, resulting in lower and more inconsistent clearance rates, as reported in various studies [44, 45]. Professional tattoos required more sessions and demonstrated reduced responsiveness compared with amateur tattoos, likely reflecting higher ink density and deeper pigment deposition [4, 45]. Colored pigments, particularly green, yellow, and red inks, showed inconsistent responses across laser platforms, underscoring the challenges associated with multicolored tattoos [6, 44, 46, 47].

Nd:YAG lasers were generally associated with a favorable safety profile, including in cohorts with darker Fitzpatrick skin types, and were associated with lower rates of pigmentary adverse effects than shorter‐wavelength laser systems in comparative studies, which may relate to the deeper penetration and relative melanin sparing of the 1064‐nm wavelength [5, 32, 48]. However, comparative safety advantages should be interpreted cautiously, given study limitations [16, 46, 49].

Transient pigmentary alterations were among the most frequently reported adverse events across studies, underscoring the importance of cautious parameter selection, appropriate treatment intervals, and patient counseling regarding potential risks, particularly in individuals with higher Fitzpatrick skin types [5, 15, 32].

The overall certainty of evidence was rated as low to very low using the GRADE framework. This reflects the predominance of observational studies and case series, small sample sizes, inconsistent outcome measures, and variable reporting of laser parameters and adverse events [3, 9]. Many studies lacked blinded outcome assessment or long‐term follow‐up, limiting evaluation of sustained clearance and delayed complications [7, 23, 26]. Additionally, clearance was assessed using non‐standardized, often subjective scales, further complicating comparisons across studies [10, 38, 50].

These limitations indicate that reported clearance rates should be interpreted as approximate estimates rather than precise predictors of individual patient outcomes [11, 36]. Publication bias cannot be excluded, given the predominance of single‐centre studies and the limited reporting of treatment failures or null outcomes [3, 9, 12]. This systematic review synthesizes up‐to‐date clinical evidence on Nd:YAG laser tattoo removal, drawing evidence across nanosecond and picosecond systems, accelerated techniques, and diverse tattoo types and patient characteristics. Collectively, the evidence supports Nd:YAG lasers as the preferred first‐line treatment for black and blue tattoos across skin types [4, 5, 23], while also underscoring uncertainty around optimal treatment parameters [34, 35], the true clinical benefit of picosecond technology [7, 13, 25] and accelerated or multipass approaches [37, 39].

Despite these limitations, the consistency of directional findings supports the continued use of Nd:YAG lasers as a first‐line modality for tattoo removal, particularly for black and blue pigments and in patients with darker skin phototypes [5, 23, 32, 49]. Clinicians should anticipate the need for multiple treatment sessions and individualize treatment parameters based on tattoo characteristics and patient factors, while counseling patients regarding realistic expectations and potential adverse effects [11, 44].

Future research should prioritize well‐designed randomized controlled trials with standardized clearance metrics, transparent reporting of laser parameters, and adequate follow‐up [3, 9, 39]. Comparative studies evaluating picosecond and nanosecond Nd:YAG systems across diverse pigment colors and skin phototypes are particularly needed [13, 25, 51]. Creating consensus outcome measures would substantially improve comparability and facilitate future quantitative synthesis.

## 5 Conclusion

Nd:YAG lasers are associated with meaningful clearance of black and blue tattoos and demonstrate a generally favorable safety profile across a range of skin phototypes. They remain the first‐line laser modality for dark tattoo pigments, although clearance typically requires multiple treatment sessions and varies according to tattoo characteristics and treatment parameters. Picosecond Nd:YAG systems may reduce treatment burden, but clear superiority over nanosecond systems has not been established. Well‐designed comparative studies with standardized outcome measures are needed to define optimal treatment strategies.

## Author Contributions

All authors S.K., J.J.E.W., M.T., Z.D., I.S. have read and approved the final manuscript. J.J.E.W. and M.T. performed the literature search. I.S. designed the research study. S.K., J.J.E.W., M.T., and Z.D. analysed the data. S.K., J.W., M.T. and Z.D. contributed to writing the paper.

## Conflicts of Interest

The authors declare no conflicts of interest.

## Supporting information


**Appendix S1:** Search strategy (MEDLINE and EMBASE via Ovid).

**Table S1:** Risk of bias assessment.

## Data Availability

Data sharing not applicable to this article as no datasets were generated or analysed during the current study.
