# Supplementary material for: Efficacy of Nd:YAG Lasers for Tattoo Removal: Systematic Review of Clinical Outcomes, Clearance Rates, and Treatment Parameters
Source: J Cosmet Dermatol. 2026 Jun 17;25(6):e70914. doi: 10.1111/jocd.70914 (PMC13276302; doi:10.1111/jocd.70914)
Supplement: Supplementary file 1 — Appendix S1: Search strategy (MEDLINE and EMBASE via Ovid). [file JOCD-25-e70914-s001.docx]

**Supplementary Appendix 1. Search strategy (MEDLINE and EMBASE via Ovid)**

**Date searched:** 11 December 2025

**Platform:** Ovid

(Nd:YAG OR Q-switched OR picosecond OR exp laser/)

AND

(tattoo* OR "tattoo removal")

AND

(wavelength* OR pulse duration* OR fluence* OR "spot size*")

AND

(clearance* OR outcome* OR adverse effects* OR pigmentation* OR scarring*)

###

###

###

###

###

###

### 
