# Supplementary material for: Efficacy of Nd:YAG Lasers for Tattoo Removal: Systematic Review of Clinical Outcomes, Clearance Rates, and Treatment Parameters
Source: J Cosmet Dermatol. 2026 Jun 17;25(6):e70914. doi: 10.1111/jocd.70914 (PMC13276302; doi:10.1111/jocd.70914)
Supplement: Supplementary file 2 — Table S1: Risk of bias assessment. [file JOCD-25-e70914-s002.docx]

# **Supplementary Table 1. Risk of bias assessment**

| Study (Author, Year) | Study Design | ROB Tool | Overall ROB | Key Sources of Bias |
| --- | --- | --- | --- | --- |
| Kilmer et al., 1993 | Prospective controlled study | RoB 2 | Some concerns | Unclear randomization |
| Leuenberger et al., 1999 | Prospective split-tattoo | RoB 2 | Some concerns | Allocation unclear |
| Ross et al., 1998 | Comparative trial | RoB 2 | Some concerns | Small sample |
| Goyal et al., 1997 | Prospective paired comparison | RoB 2 | Some concerns | Limited blinding |
| Pinto et al., 2016 | Randomized controlled trial | RoB 2 | Low | Minor imprecision |
| Narayanan et al., 2022 | Randomized split-tattoo | RoB 2 | High | Attrition bias |
| Lorgeou et al., 2018 | Randomized comparative | RoB 2 | Some concerns | Short follow-up |
| Kono et al., 2020 | Prospective comparative | RoB 2 | Some concerns | Limited power |
| Bernstein & Civiok, 2013 | Prospective split-tattoo | RoB 2 | Some concerns | Small cohort |
| Bäumler et al., 2022 | Prospective split study | RoB 2 | Some concerns | Pigment heterogeneity |
| Karsai et al., 2008 | Retrospective cohort | NOS | High | Selection bias |
| Mungnirandr et al., 2011 | Retrospective cohort | NOS | High | No confounder control |
| Jow et al., 2010 | Retrospective cohort | NOS | High | Compliance confounding |
| Egozi et al., 2024 | Retrospective cohort | NOS | Moderate | Retrospective design |
| Cannarozzo et al., 2021 | Retrospective cohort | NOS | Moderate | Variable settings |
| Sravan et al., 2023 | Interventional cohort | NOS | Moderate | No comparator |
| Nguyen et al., 2021 | Prospective cohort | NOS | Moderate | Selective population |
| Lakshmi et al., 2015 | Prospective cohort | NOS | Moderate | No adjustment |
| Padhiar et al., 2019 | Prospective cohort | NOS | Moderate | Small sample |
| Gold, 2009 | Case series | JBI | Moderate | No comparator |
| Aurangabadkar et al., 2019 | Case series | JBI | Moderate | Descriptive only |
| Hindy, 2020 | Case series | JBI | Moderate | Single-center |
| Bennardo et al., 2021 | Case series | JBI | Moderate | Selection bias |
| Alster & Kauvar, 2015 | Abstract | JBI | High | Incomplete methods |
| Abbas et al., 2021 | Case series | JBI | High | Non-standard outcomes |
| Reilly et al., 2023 | Cohort | NOS | Moderate | Social confounding |
| Wai et al., 2006 | RCT | RoB 2 | Some concerns | Adjunctive therapy |
| Kaminer et al., 2020 | Trial | RoB 2 | Some concerns | Limited blinding |
| Bernstein et al., 2015 | Trial | RoB 2 | Some concerns | Small sample |
| Zhang et al., 2018 | Comparative | RoB 2 | Some concerns | Single-center |
| Kato et al., 2020 | Cohort | NOS | Moderate | Combination protocol |
| Sirithanabadeekul et al., 2022 | Cohort | NOS | Moderate | Short follow-up |
| Biesman & Costner, 2017 | Trial | RoB 2 | Some concerns | Adjunctive device |
| Pawar et al., 2024 | Cohort | NOS | Moderate | Small sample |
| Mayada et al., 2024 | Prospective cohort | NOS | Moderate | Single pigment |
| Kauvar et al., 2017 | Trial | RoB 2 | Some concerns | No long-term follow-up |
| Boehncke et al., 1994 | Split-tattoo | RoB 2 | Some concerns | Old technology |
| Ahčan et al., 2013 | Prospective cohort | NOS | Moderate | Descriptive |
| Sardana et al., 2015 | Split-lesion | RoB 2 | Some concerns | Combination therapy |
| El-Domyati et al., 2019 | Prospective cohort | NOS | Moderate | Histologic surrogate |
| Vangipuram et al., 2018 | Prospective cohort | NOS | Moderate | Adjunctive patch |
| Gollamudi et al., 2024 | Comparative | RoB 2 | Some concerns | Single-center |
| Priya et al., 2018 | Prospective cohort | NOS | Moderate | Dermatoscopic outcomes |
| Mungnirandr et al., 2012 | Program-based cohort (Thai student removal program; regional comparison) | NOS | Moderate | Program-based selection; limited confounder adjustment; variable follow-up/reporting |
| Ross et al., 2016 (NCT02244554) | Trial registry record (pico vs nano Nd:YAG comparison) | JBI | High | Registry/unpublished record; incomplete methods and outcomes limit ROB appraisal |
| Kossida et al., 2012 | Prospective clinical study (accelerated repeated-exposure technique) | RoB 2 | Some concerns | Non-standard protocol; blinding/allocation details unclear; heterogeneity in outcome reporting |
